# Comparison of Colposcopy and Histopathology in Abnormal Cervix

**DOI:** 10.7759/cureus.54274

**Published:** 2024-02-15

**Authors:** Mangesh G Kohale, Anupama V Dhobale, Kajal Hatgoankar, Sweta Bahadure, Akshay H Salgar, Gulshan R Bandre

**Affiliations:** 1 Pathology, Datta Meghe Medical College, Datta Meghe Institute of Higher Education and Research (Deemed to be University), Nagpur, IND; 2 Obstetrics and Gynaecology, Datta Meghe Medical College, Datta Meghe Institute of Higher Education and Research (Deemed to be University), Nagpur, IND; 3 Preventive Medicine, Government Medical College and General Hospital, Baramati, IND; 4 Microbiology, Jawaharlal Nehru Medical College, Datta Meghe Institute of Higher Education and Research (Deemed to be University), Wardha, IND

**Keywords:** abnormal cervix, cervical intraepithelial neoplasia, cervical cancer, histopathological findings, colposcopy

## Abstract

Cervicovaginal cancer (CVC) is the most common malignancy of the genital tract. Colposcopy is a diagnostic procedure that is used to examine the cervix and tissue samples of the urethra and vulva in a magnified view. The colposcopy and histological findings of unhealthy cervixes in a tertiary care hospital were compared. This comparative cross-sectional study was conducted from November 2022 to March 2023 among women with a variety of gynecological complaints who visited the Department of Obstetricians and Gynecologists in a tertiary care facility. One hundred participants were included in this study. The data collected were analyzed using Microsoft Excel 2016 (Microsoft Corporation, Redmond, Washington, United States). Participants had an average age of 35.22 ± 7.18 years, and white discharge was the most reported ailment (73%). Comparing the results of the colposcopy with the histological findings revealed a sensitivity of 91.5% and a specificity of 72.2%. The high sensitivity of colposcopy highlights the need to combine it with histological techniques to obtain better results.

## Introduction

Cervical cancer is a major health issue that affects women around the world [1]. Premalignant lesions in the transformation zone of the uterine cervix (where the epithelial and columnar epithelia meet) are distinguished by the cellular or epithelial architecture [2]. In India, in 2022, cervical cancer represented 9% (127,526) of all cancer cases and 17.7% of all female cancers [3]. In 2022, 662,301 new cases of cervical cancer were reported globally, and there were 348 874 deaths [4].

However, screening efforts have dramatically reduced cervical cancer rates worldwide, with the developing world accounting for 80% of all deaths related to cervical cancer [2]. The incidence and prevalence of cervical cancer are still high in developing countries due to the lack of screening programs. Colposcopy is a diagnostic procedure consisting of examining samples of cervix and vulvar tissue in a magnified view. Numerous premalignant and malignant tumors in these regions can be identified by colposcopy due to their distinct characteristics. The colposcope is useful for observing the cervix, differentiating between normal and abnormal areas, and performing direct surgeries based on abnormal changes for additional pathological analysis. The primary objective of colposcopy is the early detection and treatment of precancerous lesions to prevent cervical cancer [5]. As it is a lengthy pre-invasive stage, cervical cancer can be prevented by early detection and access to screening tests [6].

The aim of this study is to correlate colposcopy and histological findings of unhealthy cervixes in a tertiary care hospital and examine the results of a cervical colposcopy in poor health.

## Materials and methods

Study design and participants

This comparative cross-sectional study was conducted in the Department of Obstetrics and Gynecology, Datta Meghe Medical College, Nagpur, Maharashtra, India, from November 2022 to March 2023, after approval of the Institutional Ethics Committee of Shalinitai Meghe Hospital & Research Centre, Nagpur (approval number: SMHRC/IEC/2022/09-39). Participants were selected based on specific criteria: having an unhealthy cervix on pelvic examination, recurrent vaginitis, postcoital, or both. Patients who had previously been diagnosed with cervical malignancy or received treatment were excluded from the study.

Sample size determination

The sample size of 100 participants was calculated using PASS software (NCSS, LLC, Utah, United States), employing a predetermined methodology.

Data collection

Data were collected by documenting the results of colposcopy and histology reports. Abnormalities during colposcopy, including the presence of acetowhite areas and abnormal vessels, were considered abnormal findings, regardless of their severity. Subsequently, all participants underwent a biopsy for histopathological evaluation. Histological changes were classified as normal, CIN1, CIN2, CIN3, or invasive carcinoma. Patients with human papillomavirus (HPV)-related changes or other borderline conditions were not included in the study.

Data analysis

The data collected were analyzed using Microsoft Excel 2016 (Microsoft Corporation, Redmond, Washington, United States). Percentages and proportions were used to summarize the findings.

## Results

Table 1 describes the age profile of the patients. Of 100 patients, the majority of participants (57%) fall within the age range of 31-40 years. Twenty-three (23%) belong to the 41-50 age group. The distribution becomes progressively smaller in the older age categories, with nine (9%) in the 51-60 age group and four (4%) among participants older than 60 years. The youngest age group (≤30) comprises 7 (7%) cases of the total sample.

**Table 1:** Age distribution

Age group (years)	Frequency (n=100)	Percentage (%)
≤30	07	07.00%
31-40%	57	57.00%
41-50	23	23.00%
51-60	09	09.00%
>60	04	04.00%
Total	100	100%

The predominant clinical symptom in the patient cohort was white discharge, presenting in 73 (73%) cases, indicating its substantial prevalence. Intermenstrual bleeding was observed in 11 (11%) patients, signifying its occurrence in a noteworthy but smaller subset. Post-coital bleeding was reported in nine (9%) cases, underscoring its distinct clinical manifestation. Post-menopausal bleeding, identified in seven (7%) patients, suggested its emergence as a distinct clinical entity, particularly in individuals beyond the menopausal stage. Pelvic pain, while less prevalent at six (6%), remained noteworthy, indicating its presence in a specific subset of cases within the studied population (Table 2).

**Table 2:** Symptoms among patients (N=100)

Clinical symptoms	Frequency (n=100)	Percentage %
White discharge	73	73%
Intermenstrual bleeding	11	11%
Post-coital bleeding	9	9%
Post-menopausal bleeding	7	7%
Pelvic pain	6	6%

Normal colposcopic findings were noted in 20 (20%), while the most prevalent category (n=47, 47%) comprised inflammation, squamous metaplasia, or erosion. Hazy acetowhite areas were present in 13 (13%) cases, and mosaic-like changes (mosaicism) were detected in 11 (11%). Colposcopic assessments were deemed unsatisfactory in six (6%) cases, while malignant features, characterized by intense acetowhite lesions, coarse irregular punctations, and corkscrew vessels, were identified in three (3%) patients (Table 3).

**Table 3:** Colposcopy findings (N=100)

Colposcopy findings	Frequency	Percentage
Normal	20	20%
Inflammation/squamous metaplasia/erosion	47	47%
Hazy/faint acetowhite areas	13	13%
Mosaicism	11	11%
Unsatisfactory	06	6%
Malignancy (intense acetowhite lesion, coarse irregular punctations, corkscrew vessels)	03	3%

The specificity of 72.22% underscores colposcopy's accuracy in correctly identifying true negatives, primarily in cases with normal findings. The positive predictive value of 93.75% signifies a high probability that cases identified as abnormal by colposcopy are indeed abnormal, while the negative predictive value of 65% indicates a moderate probability that cases identified as normal by colposcopy are truly normal. The overall accuracy of colposcopic findings compared to histopathological results is 88%, highlighting its effectiveness in providing correct diagnostic outcomes (Table 4).

**Table 4:** Correlation of colposcopy with gold standard histopathological findings

Colposcopy findings	Histopathological findings	
	Abnormal (%)	Normal (%)
Abnormal	75 (91.46)	05 (27.78)
Normal	07 (08.54)	13 (72.22)
Total	82	18

In evaluating cervical health, the colposcopy demonstrated a sensitivity of 91.46% (95%CI: 83.20-96.50) and specificity of 72.22% (95%CI: 46.52-90.31). The positive likelihood ratio was 3.29 (95%CI: 1.56-6.96), while the negative likelihood ratio was 0.12 (95%CI: 0.06-0.25). With a disease prevalence of 82.00%, the positive predictive value was 93.75% (95%CI: 87.66-96.94%), and the negative predictive value was 65.00% (95%CI: 46.38-79.95%). The overall accuracy of the colposcopy was 88.00% (95%CI: 79.98-93.64%) (Table 5).

**Table 5:** Statistical findings of colposcopy

Statistic	Value	95% CI
Sensitivity	91.46%	83.20-96.50
Specificity	72.22%	46.52-90.31
Positive likelihood ratio	3.29	1.56-6.96
Negative likelihood ratio	0.12	0.06-0.25
Disease prevalence	82.00%	73.05-88.97
Positive predictive value	93.75%	87.66-96.94
Negative predictive value	65.00%	46.38-79.95
Accuracy	88.00%	79.98-93.64

## Discussion

Abnormal cervix includes chronic cervicitis, including endocervicitis, cervical erosions, lacerations, and leukoplakia. Even if the Pap smear is negative, these lesions can be premalignant. Most early-stage cancers have no symptoms [7]. Therefore, after histological analysis of biopsies obtained during colposcopic examination or from a grossly abnormal cervix, a diagnosis is typically made. Cervical invasive cancer is considered a preventable condition because it has a lengthy pre-invasive stage, which makes it amenable to screening and treatment. Routine screening and HPV vaccination are recommended to reduce the future prevalence of cervical cancer [8]. In developing countries such as India, cytology-based screening programs have had very little success due to a lack of qualified personnel, laboratory space and equipment, high service costs, and inadequate follow-up. Therefore, it is necessary to identify an alternative screening method like cytology screening (Pap test), visual inspection tests like 3-5% acetic acid (VIA) and Lugol’s iodine (VILI), and HPV polymerase chain reaction (PCR) [9].

The mean age of the patient in the current study was 35.22 ±7.18 years (Table 1). In a study on the evaluation of an unhealthy cervix, Gohil et al. observed that most patients (53.33%) were over 40 years [10]. Similar results were found in studies by Upadhyay et al. [11] and Joshi et al. [12], in which the mean age of patients was 36.4 and 32.2 years, respectively. Other studies by Pimple et al. [13] and Boicea et al. [14] have reported similar patient distributions. White discharge was the most common (73%) symptom, followed by irregular periods of blood loss (11%), post-coital hemorrhage (9%), and postmenopausal bleeding (7%) (Table 2). Savitha et al. also observed that white discharge per vagina was the most common symptom (86%) among cases [15]. Similar findings were reported by Upadhyay et al. [11], Chaudhary et al. [16], and Bhalerao et al. [17]. Most of the patients had findings of inflammation (47%) followed by acetowhite areas (13%). Malignancy was observed in 3% of the patients, with normal findings in 20% (Table 3).

In a study on the evaluation of an unhealthy cervix, Gohil et al. found that 50% of the population had no abnormality, 32.5% had acetowhite areas, 5% had mosaicism, 2.5% had punctures, and 1.6% had atypical vessels [10]. Among female colposcopies, 8.33% were unsatisfactory. Interestingly, all patients with subpar colposcopy had an atrophic cervix. In colposcopy, Zainab et al. found that 55.76% of females had squamous metaplasia, 22.11% had affective zones, 10.57% had perfect punctuation, 9.61% had abrasive punctures, and 2% had mosaicism [18]. Joshi et al. reported that 27% of colposcopies were normal, 43% had acetowhite areas, 16% had punctuation, and 14% had mosaicism [12]. Vidhyadhar et al. reported that 3.8% had unsatisfactory colposcopy while 51.9% had normal colposcopy [19]. According to Gandi et al., 54% had standard observations, 31% had lower-grade lesions, 12% had high-grade or mysterious malignancies, and 3% had unsatisfactory colposcopy [20].

Savitha et al. also observed inflammation (60%) as the most abnormal finding on colposcopy [15]. This finding is consistent with the results of the present study. The current study demonstrates responsivity of 91.5%, selectivity of 72.2%, positive predictive value of 93.7%, negative predictive value of 65%, and precision of 88% once compared to histological findings. In the study by Gohil et al., they observed a sensitivity of 87.87% and a specificity of 72.72% [10]. Savitha et al. reported that the sensitivity and specificity of colposcopy were 85% and 83.75%, respectively [15]. Upadhyay et al. reported that the sensitivity and specificity of colposcopy were 94.1% and 87.8%, respectively [11]. Mazihah et al. reported an accuracy rate of 94% [21], and Ashmita et al. reported 86.54% [22]. Compared to our study, Mazihah et al. [21] and Upadhyay et al. [11] had higher sensitivity, and Savitha et al. [15] and Ashmita et al. [22] had lower values. Although the primary purpose of colposcopy is to determine where to perform a biopsy, it also helps to improve the identification of intraepithelial lesions.

HPV screening by PCR is costly hence most of the time screening with colposcopy and histopathology can be an alternative and cost-effective method [23]. When detected in the precancerous stage, cervical cancer is one of the diseases that is highly preventable and highly treatable. The fact that a vast majority of people are unaware of the disease and that India lacks a formal screening program is one of the biggest issues the country faces [24]. It takes at least 10 years for preinvasive cervical cancer to progress to invasive carcinoma. Therefore, the incidence of death related to cervical cancer can be reduced by adequately screening for cervical cancer. Numerous screening techniques are available, including Pap smears, colposcopies, visual inspection of acetic acid, and HPV, deoxyribonucleic acid testing [25]. HPV DNA testing is considered a highly effective and sensitive screening method. While Pap smears and colposcopies are valuable, HPV DNA testing offers superior accuracy in detecting high-risk strains, contributing to more efficient and cost-effective cervical cancer screening [26,27].

The findings of the present study support the notion that the addition of screening tests, as opposed to a single test, can maximize the early cervical cancer diagnosis in women. As is often said, prevention is preferable to treatment [28]. The strict implementation of screening tests and increasing awareness of the side effects of human diseases and available treatments are essential to reduce the overall incidence of reported deaths due to cervical cancer [29].

Limitations of the study

Despite the valuable insights provided by this comparative cross-sectional study on the correlation between colposcopy and histopathological examination in cervical health, certain limitations should be acknowledged. Firstly, the study's single-center design at a tertiary care facility may restrict the generalizability of its findings to a broader population, as healthcare practices and patient demographics can vary across different settings. The relatively small sample size of 100 participants, although determined statistically, might limit the study's ability to capture the full spectrum of cervical health variations. Additionally, the exclusion of patients with a prior diagnosis or treatment history may introduce selection bias, potentially excluding cases crucial for a comprehensive understanding of cervical abnormalities. The absence of detailed information on ethnic and socioeconomic backgrounds further hinders a nuanced analysis of how these factors may influence cervical health. Moreover, the retrospective nature of data collection may be susceptible to incomplete medical records or variations in data quality. Finally, the study's dependence on operator-dependent techniques like colposcopy and histopathology introduces the possibility of inter-observer variability, impacting the consistency of results. Addressing these limitations in future research will contribute to a more robust understanding of the correlation between colposcopy and histopathological examination in diverse populations and healthcare settings.

## Conclusions

This comparative study underscores the importance of combining colposcopy with histological examination for accurate diagnosis in cervicovaginal cancer cases. The high sensitivity of colposcopy, at 91.5%, highlights its efficacy in identifying abnormalities, but the specificity of 72.2% emphasizes the need for supplementary histological analysis. The study contributes valuable insights into the correlation between colposcopy and histopathology, emphasizing the significance of a comprehensive approach to cervical health assessment. Further research with larger and more diverse populations is warranted to enhance the generalizability of findings and address the study's limitations.
